# Analysis of the pressure requirements for silk spinning reveals a pultrusion dominated process

**DOI:** 10.1038/s41467-017-00409-7

**Published:** 2017-09-19

**Authors:** James Sparkes, Chris Holland

**Affiliations:** The Natural Materials Group, Department of Materials Science and Engineering, The University of Sheffield, Sir Robert Hadfield Building, Mappin Street, Sheffield, South Yorkshire UK

## Abstract

Silks are remarkable materials with desirable mechanical properties, yet the fine details of natural production remain elusive and subsequently inaccessible to biomimetic strategies. Improved knowledge of the natural processes could therefore unlock development of a host of bio inspired fibre spinning systems. Here, we use the Chinese silkworm *Bombyx mori* to review the pressure requirements for natural spinning and discuss the limits of a biological extrusion domain. This provides a target for finite element analysis of the flow of silk proteins, with the aim of bringing the simulated and natural domains into closer alignment. Supported by two parallel routes of experimental validation, our results indicate that natural spinning is achieved, not by extruding the feedstock, but by the pulling of nascent silk fibres. This helps unravel the oft-debated question of whether silk is pushed or pulled from the animal, and provides impetus to the development of pultrusion-based biomimetic spinning devices.

## Abstract (web summary)

The natural production of silks remains elusive and subsequently inaccessible to biomimetic strategies. Here the authors show that silks cannot be spun by pushing alone, and that natural spinning is dominated by pultrusion, which provides design guidelines for future biomimetic spinning systems.

## Introduction

The ability to artificially produce silk fibres has great commercial, industrial and scientific implications. Much has been made of natural silk’s remarkable mechanical properties^1–5^, but few have considered how they are imparted onto the initially liquid silk feedstock during the spinning process^6^. This study attempts to bridge this gap in our knowledge by exploring how internal pressure affects fibre production. Such efforts are important, both in terms of understanding the flow conditions in natural silk spinning and as design criteria for larger-scale manufacture of both bioinspired products^7, 8^ and biomimetic fibres^1^.

Silks are a family of natural protein biopolymers produced by spiders, silkworms and many other arthropods^9–13^. They are used in a multitude of environments, for many different tasks, including building cocoons, lining burrows, catching and preserving prey, and as swaddling cloth for their offspring^10^. Yet unlike other biological materials, they are defined as being spun rather than grown, with spinning taking place at the point of delivery^2, 6, 14, 15^.

Although silk can be (and is) reeled from live animals, it is time, space and labour intensive, making industrial upscaling difficult^9, 15–26^. Through forced reeling of *Bombyx mo*ri silkworms, fibres can be extracted whose mechanical properties are much closer to those exhibited by spider dragline silks^1,22, 27, 28, 29^, suggesting that processing, as well as the feedstock, plays a major role in silk fibre properties^26, 30^. In this study, we will focus on the use of *B. mori* as a reference system, due to the breadth of knowledge surrounding this species, and the existing commercial market for silkworm silks.

**Fig. 1:**
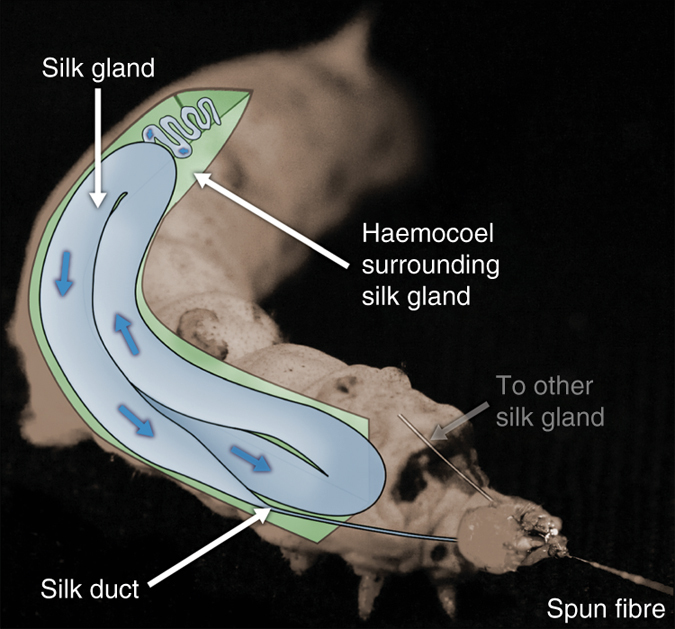
Anatomical overview of the *B. mori* silk gland. Diagram depicting the silk spinning apparatus of *B. mori*, highlighting the gland (*blue*) surrounded by the haemocoel (*green*). Direction of silk flow during spinning is indicated by the *blue arrows*

Prior to spinning, silk is stored inside the animal as a concentrated aqueous protein solution in a specialised organ known as the silk gland^2, 14^ (Fig. 1). In the rear of the gland, the primary component is fibroin, which forms the solid core of the resultant fibres. Fibre formation is the result of fibroins flowing along the gland^31^, being coated in successive layers of additional proteins^32^—sericins—before reaching a tapering duct where fibrillation is thought to be induced through a combination of changes in both pH and ionic environment^2, 31, 33–39^, and mechanical shear^10, 33, 40, 41^, arriving as a fibre when exiting the animal. However, the mechanism by which this flow occurs is not fully understood, and the roles of geometry^15, 42, 43^ and chemical changes have only been briefly explored^19, 29, 34–39, 44–46^.

There have been multiple attempts to create silk protein feedstocks^47–49^, but they have generally been frustrated by problems with molecular length^50^, aggregation^51^ and concentration^52^, with the fibres produced so far being comparable to synthetic textiles^1, 29^. We believe that this is in part due to not fully understanding how nature processes a silk feedstock, and that this is key to successfully handling these wilful proteins. Over the past decade there have been numerous studies on silk flow^4, 29, 37, 44, 49, 53–56^, but the recent publication of extensive silk rheology data^57–60^ allows us to make progress in this direction. Therefore one of the further aims of this work is to address the limitations highlighted in previous silk flow models.

Although several studies have used molecular dynamics simulations^61–68^ to explore the flow and aggregation of silk at a molecular level, and despite pipe flow being a well-established engineering approach^69^ to explore the flow of fluids in tubes, previous attempts to model silk flow at a microscale are described in just three studies. Kojic et al.^70^ made the first geometric approximations of silk spinning ducts, but although industrially relevant, the geometry used bears little resemblance to the biological system, and was limited by the absence of high quality rheological data. Moriya et al.^40^ produced a three-dimensional reconstruction from microtomography, but the complexity of the reported geometry would be extremely challenging to replicate industrially. Breslauer et al.^43^ improved on this by using Asakura et al.'s^71^ approximation of the tapering duct as a hyperbolic function (industrially relevant), but was reliant on the same limited rheological data employed by Kojic et al.^70^.

Whilst significant progress has been made in the acquisition of robust rheological data for *B. mori* ^57–60^, the geometric aspect has rarely been considered, a notion which provides us with the impetus for this work. Recent work has shown that the spinning ducts of both *B. mori* and *Nephila clavipes* are reinforced using chitin in a manner which suggests that they will be less readily deformable than the initial part of the glands, and that precisely defined geometry may be important for efficient and effective fibre spinning^42, 72^. The use of mathematical functions rather than data extracted from individual specimens allow control over this geometry in silico, which can be used to systematically explore the effect of geometric variation on the pressure requirements. It is hoped that by considering the pressure required to induce flow, this study will go some way towards answering the oft-debated question of how silk flows—is it pushed, or is it pulled?

To resolve this issue and define silk’s processing space, finite element (FE) simulations have been carried out in COMSOL Multiphysics 5.2 to explore the internal pressure required to generate flow at a given rate through a range of geometries. By treating inlet pressure as the unknown variable rather than as a known parameter as in previous studies^40, 43, 73^, we can explore the full range of pressures required for the known physiological spinning speeds of *B. mori*. Experimental validation was undertaken through a practical fibroin extrusion apparatus (pushing) and the application of a forced reeling technique (pulling), both of which support our simulations and seek to define the physical limits of natural spinning and guide the future development of biomimetic spinning devices. We report that the pressure required for flow is significantly higher than silk producers can generate in their haemolymph, and conclude that rather than being an extrusion based system, the process of silk spinning is dominated by pultrusion effects, which act via the solidity gradient induced as fibrillation occurs.

## Results

### Comparison of existing silk geometries

**Fig. 2:**
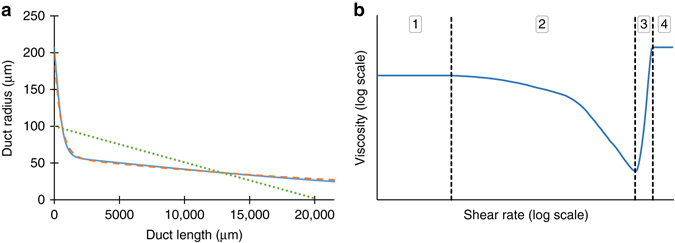
Geometric and rheological considerations. **a** Previous geometric approximations of silk ducts showing the variation in radius as a function of distance along the duct. *Curves* represent Asakura (*orange dashes*) and Breslauer’s (*blue line*) hyperbolic approximations, along with the linear model employed by Kojic (*green dots*). The equations describing each curve can be found in Supplementary Table 1. **b** – Exemplar rheological response of silk feedstock to increasing shear rate, showing four distinct regimes - (1) – Pseudo-Newtonian behaviour at low shear rates; (2) – Shear thinning behaviour; (3) – Fibrillation, increasingly elastic response; (4) – Viscoelastic solid, second plateau reached

There have only been three previous studies which have modelled the flow of silk, of which only two used geometries that can be considered industrially applicable. Two were described by a hyperbolic function (Asakura et al.^71^ and Breslauer et al.^43^), while the third used a linear approximation (Kojic et al.^74^, Fig. 2a). Since both hyperbolic functions were derived from the same data, this effectively leaves just two geometric models. Asakura’s, with an eightfold reduction, and Kojic’s, with a fifty fold reduction from an inlet half the size of the natural system. Kojic’s outlet diameter is marginally smaller than is seen in an individual *B. mori* fibre (~10 µm), whereas Asakura’s is 2.5 times larger.

### Comparison of existing silk viscosity models

Several different generalised viscosity models have been applied to empirically measured fibroin data^4, 37, 40, 44, 54, 55, 57, 58, 73–75^ in previous studies; these can be grouped as either Newtonian^75^, or non-Newtonian^37, 40, 44, 54, 55, 57, 58, 73^. Contemporary studies are in agreement that fibroin behaves, as expected of a polymeric solution, as a non-Newtonian fluid, but the methods used to describe this behaviour vary^4, 37, 40, 44, 54, 57, 58, 73–75^. The response of silk to an increasing shear rate can be described in four distinct regimes (Fig. 2b).

The mathematical models that have been used to describe the viscoelastic behaviour of fibroin are both complex and not well defined, particularly at higher shear rates where fibre formation is known to begin. Early attempts to describe this behaviour used a different power law function to describe each of phases 1–4 in Fig. 2b ^37, 40, 44, 73^. However, as we are not yet concerned with the fibrillation region (Fig. 2b, regions 3 and 4) (a biomimetic design limit—premature fibrillation will be naturally selected against, as duct blockages would prevent further spinning), models which describe the behaviour at shear rates below this point (regions 1 and 2 in Fig. 2b) are considered adequate. As such, the Carreau-Yasuda model^76^ (Equation 1) is now the preferred model as it describes regions 1 and 2 in a single equation.

**Fig. 3:**
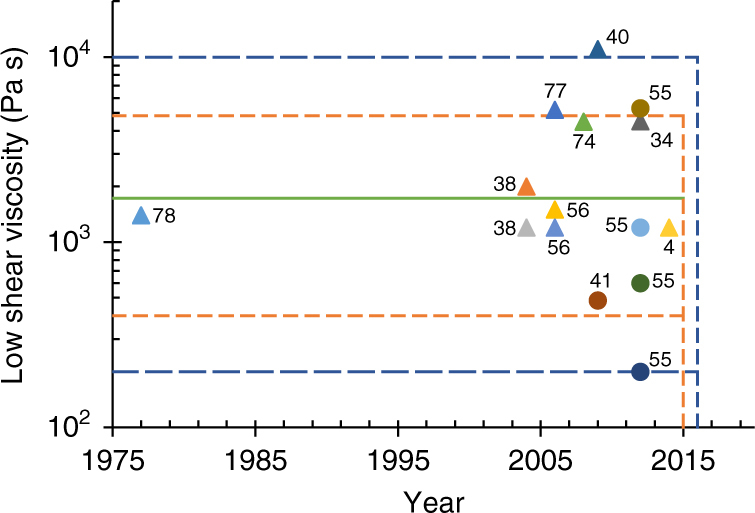
Variation in wild and domesticated silkworm feedstock viscosities. Measured values of the low shear viscosity of wild (*circles*) and domesticated (*triangles*) silkworm feedstocks from previous rheological studies^4, 33, 37, 40, 54, 55, 57, 58, 73–75^, the highlighted regions show the mean (*green line*), and ranges proposed by Laity’s 2015 (*orange dots*)^57^ and 2016 (*blue dashes*)^58^ studies (data labels provide references). This suggests that a large sample size is key to understanding the variability in the viscosity of silkworm feedstocks

The rheological properties of the silk feedstock used in the simulations are based on a range derived from literature values obtained over the past 40 years^4, 37, 40, 44, 54, 57, 58, 73–75, 77, 78^. Their proposed low shear viscosities (≤1 s^−1^) are shown in Fig. 3.

The viscoelastic properties of the fluid are described using the Carreau-Yasuda model^76^, typically written as:

1 $$\eta \left( {\dot \gamma } \right) = {\eta _\infty } + \left( {{\eta _0} - {\eta _\infty }} \right){\left( {1 + {{\left( {\lambda \dot \gamma } \right)}^a}} \right)^{\frac{{n - 1}}{a}}}$$

Here, the dynamic viscosity $\eta \left( {\dot \gamma } \right)$ is modelled as a function of shear rate $\dot \gamma$, which varies between two Newtonian plateau regions—the zero (*η*_0_) and infinite (*η*_∞_) shear viscosities—between which lies a region of non-Newtonian, shear thinning, behaviour which is curve-fitted using *a* and *n*. Note that *λ* represents the fluid’s relaxation time.

We simplify this model by assuming that since the feedstock will gel above a certain shear rate, there is no need to include the infinite shear viscosity *η*_∞_, as it does not have physical significance, and thus the model simplifies to:

2 $$\eta \left( {\dot \gamma } \right) = {\eta _0}{\left( {1 + {{\left( {\lambda \dot \gamma } \right)}^a}} \right)^{\frac{{n - 1}}{a}}}$$

The simplified form of the CY-model (Equation 2) allows the viscosity to be described in terms of its zero shear viscosity (readily estimated from low shear measurements) and the rate of shear thinning. This seems appropriate, given that the primary difference between individual specimens lies in the reported values of the low shear viscosity (Fig. 3), with successive studies showing values varying between ~800 and 10^8^ Pa s^4, 44^. Recent work on a much larger sample size^57^ shows that *B. mori* feedstocks have a range of low shear viscosities, spanning 400–5000 Pa s (mean = 1722 Pa s, s.d. = 935 Pa s, *n* = 125), which incorporates all previously reported values with one major exception^44^, which it suggests has gelled and is therefore irrelevant. This was further extended by considering viscosity as a function of percentage cocoon spun, to show that the range spans ~200–10,000 Pa s^58^. Although this pertains to *B. mori*, many other silk producers (including other silkworms and spiders) lie within this bracket^54, 55^.

### Pressure

In order to determine valid pressures it is necessary to consider the physiological limitations which constrain the environment in which we operate. The larval stages of most endopterygota maintain a positive internal pressure, which gradually reduces to atmospheric levels as sclerotisation in the adult instar increases^79^. Steady internal pressure is maintained through muscular contraction, with small variations observed to coincide with heartbeats^80, 81^. This active maintenance is readily observed during anaesthesis, whereupon internal pressure is lost and the body becomes flaccid^82^. In soft bodied, non-segmented larvae, haemolymph pressure is effectively equivalent to the internal pressure of the whole body^80^.

We define the upper limit for internal pressure in silkworms as one that induces eclosion/ecdysis (shedding of the skin). Although silkworms are clearly capable of producing such pressures, to do so ruptures the haemocoel (primary body cavity), which could prove fatal if undertaken prematurely, therefore the pressure that could be applied to the silk glands must be less than this. This assumption is considered valid due to the non-segmented nature of the haemocoel, resulting in a uniform body pressure which cannot readily be directed or localised within the haemocoel itself (Fig. 1).

**Fig. 4:**
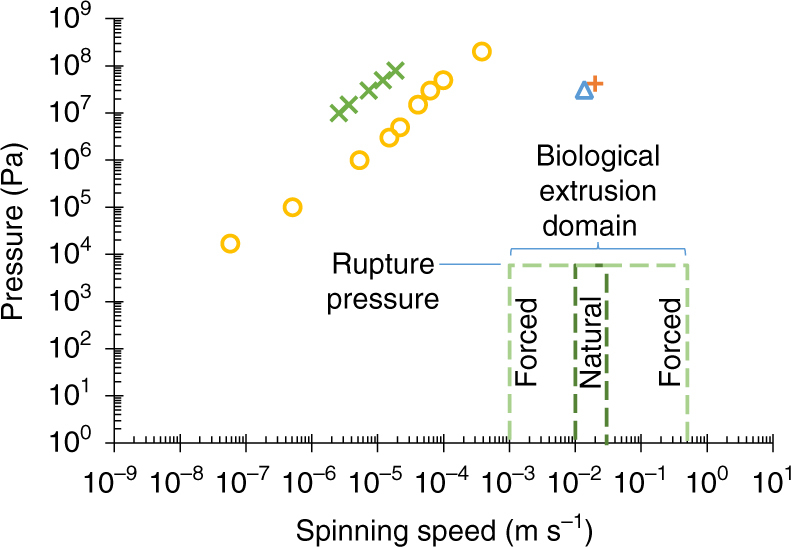
Biological relevance of previous pressure estimations. A comparison of previous estimations (Kojic (2006), *orange plus*; Moriya (2008), *yellow circles*; Moriya (2009), *green x*; Breslauer (2009), *blue triangle*) of the pressure requirements for silk spinning in* B. mori* silkworms, highlighting the discrepancies between both spinning speed and pressure compared to the natural system. Although the most recent models have aligned spinning speed, the pressure remains too high. The biological extrusion domain is split into natural (*d* *ark green dashes*) and forced (*pale green dashes*) spinning domains

Figure 4 summarises previous estimations from the literature of internal pressure, and the resulting predicted spinning speeds. To facilitate evaluation of previous studies, the following boundaries are introduced, which describe the limits of what is known as the biological extrusion domain. Although fibres may be spun outwith these constraints, they cannot be considered representative of the natural systems. An initial constraint is the range of natural spinning speeds (0.01–0.03 m s^−1^)^22, 83^, but we widen this to incorporate forced reeling (0.001–0.5 m s^−1^)^15, 21, 22, 25, 84^ — since silks can be made by the animal across this range — to provide the forced and natural biological extrusion domains.

The upper limit on pressure is the maximum recorded internal pressure in the insecta class—4 × 10^4^ Pa for hard bodied species^85^. This is reduced to a lowly 5880 Pa—the haemocoelic rupture pressure of final instar *B. mori* entering pupation^79^ (adult moths have been recorded at marginally higher internal pressures (6750 Pa)^86^, but this is not representative, since they no longer contain silk glands and have developed exoskeletons).

### Geometric effects

**Fig. 5:**
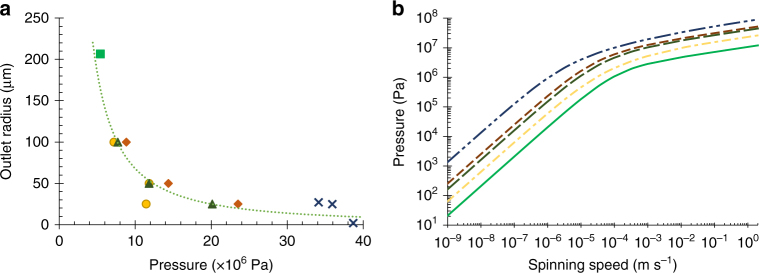
The effect of geometric variation on pressure requirements. **a** The relationship between required pressure at inlet, and duct geometry (linear (*yellow circles*), parabolic (*orange diamonds*), exponential (*dark green triangles*), zero taper (*pale green square*), and literature (*blue x*) values) at natural spinning speeds (0.02 m s^−1^). Greater and more severe tapers both increase pressure required to flow representative feedstock. **b** The effect of geometric variation on required inlet pressure. A limited, representative dataset is shown for clarity (x8 diameter reduction of parabolic (*orange dots*), exponential (*dark green dashes*), and linear tapers (*yellow dot-dashes*)), all curves lie between the limits shown above of Kojic’s model (*blue dot-dashes*), and the limiting case (zero taper,* pale green*). See Supplementary Fig. 3 for further details

The use of different duct geometries allowed the effect of shape on the flow to be assessed (Fig. 5). From a given inlet diameter (413 µm^71^), ducts were created using mathematical functions (Supplementary Tables 1 and 2) to describe both the severity of the taper, and the outlet diameter. Parabolic, exponential, and linear ducts were analysed, each with outlet diameters at ^1^/_2_, ^1^/_4_ and ^1^/_8_th of the inlet, and for the linear taper, an additional limiting case with outlet diameter equal to inlet (zero taper). It can be seen that both an increase in outlet diameter (Fig. 5a) and a reduction in the severity of the taper (Fig. 5b), serve to reduce the pressure requirements. However, even in the limiting case, the pressure required to spin at natural speeds (5.4 × 10^6^ Pa) remains many orders of magnitude higher than that recorded in *B. mori* ^79, 86^, and is clearly unrepresentative. Of further interest is that the extensional flow rates are variable along the length of the duct (Supplementary Fig. 1), which directly contradicts the suggestion that hyperbolic tapers are used to provide a constant extensional flow^87^.

### Viscosity effects

**Fig. 6:**
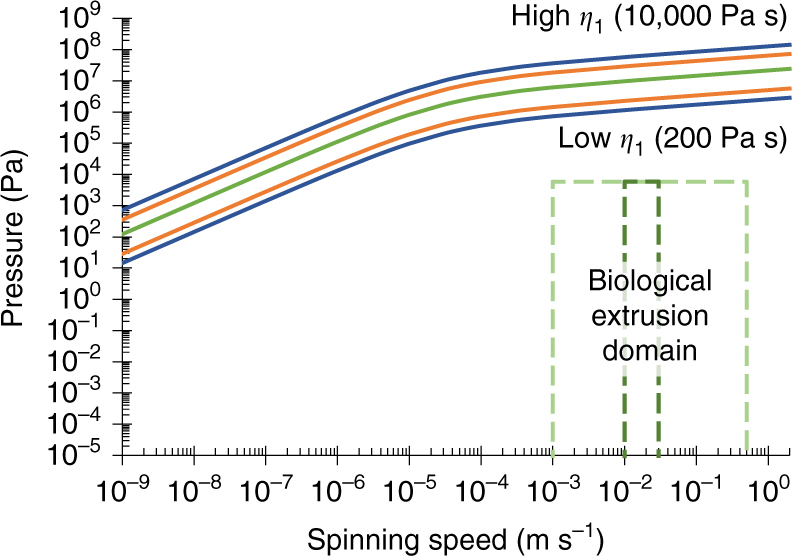
The effect of viscosity variation on pressure requirements. The effect of varying the zero shear viscosity (across the range 400–5000 Pa s^57^ (*orange*), mean (1722 Pa s^58^, *green*), and 200-10,000 Pa s^58^ (*blue*)) on the required inlet pressure. Since geometry was earlier shown to have little effect on the pressure requirements, this study employed the same geometry as Breslauer et al.^43^ to allow comparison with previously published work. The biological extrusion domain is split into natural (*dark green dashes*) and forced (*pale green dashes*) spinning domains

The variation in models using the Carreau-Yasuda method lies primarily in the value of the zero shear viscosity, with the rate of shear thinning shown to be similar across previous studies (Supplementary Fig. 2). This effectively provides a single variable whose effect can be readily explored through simulation. Figure 6 shows the effect of varying the zero shear viscosity across the range detailed in Laity’s 2015 study^57^ in relation to the biological extrusion domain. It is clear that reduced zero shear viscosity reduces the pressure required for spinning, yet silk feedstocks across this range of viscosities are all viable spinning feedstocks in vivo, suggesting that zero shear viscosity has little effect on the spinning process. However, reducing zero shear viscosity alone is clearly not sufficient to enter the biological extrusion domain, and thus factors must play a role in reducing the pressure required to spin.

### The effect of wall friction

**Fig. 7:**
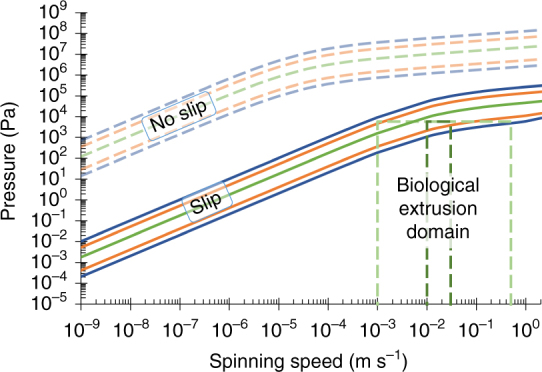
The effect of wall boundary conditions on pressure requirements. The assumption of a slip condition at the duct wall (with the same geometric and rheological data as used in Fig. 6) brings the lower portion of Laity’s ranges (*green*: mean-1722 Pa s^57, 58^, *orange*: 400-5000 Pa s^57^, *blue*: 200-10,000 Pa s^58^) into the biological extrusion domain. The biological extrusion domain is split into natural (*dark green dashes*) and forced (*pale green dashes*) spinning domains

The effect of reducing friction at the wall of the duct, resulting in reduced pressure requirements, can be clearly seen in Fig. 7. Reduction of wall friction is a common theme in pipeline networks (e.g., for oil) via surface modification^88, 89^ or addition of drag reducing agents^90^, yet even in the limiting case for *B. mori* where the wall is considered to be at 100% slip, the pressure does not drop sufficiently far across the biological range of feedstock viscosities to entirely enter the spinning domain.

At the far end of minimising the interaction between feedstock and duct wall is the possibility of a lubricating layer to minimise friction. Many authors have suggested that there may be some form of lubrication present in silk ducts^31, 55, 71, 91–94^. Of the ~80 different proteins identified in silk feedstocks^95^, most opt for it being a secondary function of sericin, though there is little evidence to support this. Brown et al.^91^ suggest that unbound water may perform this role, while data originally dismissed as erroneous due to phase separation^96^ (resulting in a reduction in force measured) in Holland’s 2006 study^55^, and the layering effects seen in 2012^33^, actually strengthen this suggestion. A simplistic approximation is to assume that if this is the case, then plug flow can be assumed, which allows us to determine the pressure requirements by modelling the flow as solely consisting of a lubricant.

### Changing the fluid

**Fig. 8:**
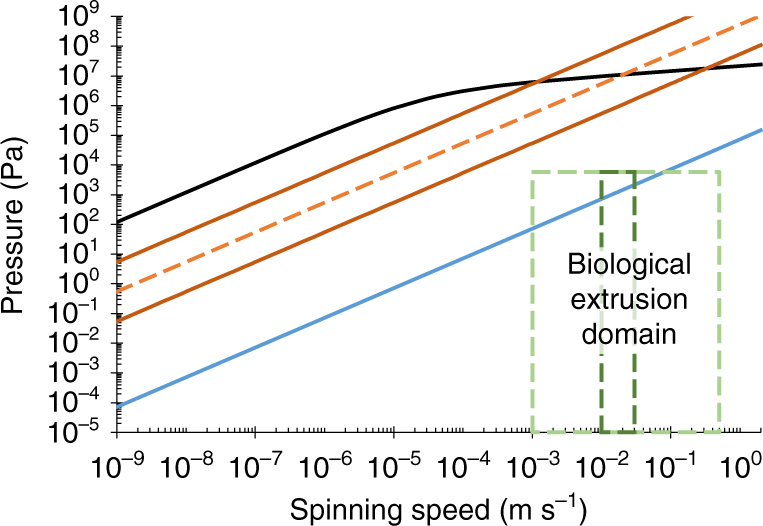
The effect of using an alternative working fluid on pressure requirements. The use of lower viscosity fluids (in the same geometry used by Breslauer with a no-slip wall condition) reduces the pressure required compared to fibroin (*black*: *η*_0_ =  1700 Pa s) when the working fluid is sericin (*orange dashes*:  *η*_0 _= 7.6 Pa s; *orange line*: ± order of magnitude variation), or water (*blue*: *η* = 8.9 x10^−4^ Pa s). Water only just reaches the biological extrusion domain, with the higher natural spinning speeds remaining unattainable. The biological extrusion domain is split into natural (*dark green dashes*) and forced (*pale green dashes*) spinning domains

If one considers the case where the interaction between fibroin and wall of the duct is minimised, wherein it still appears impossible to reach the biological extrusion domain, then another option is to focus our attention on the use of other fluids in lieu of the non-Newtonian fibroin. It is well known that in *B. mori*, although the glands are considered to be primarily composed of fibroin, there also exists a relatively large proportion of sericin, which forms up to 30% of the final fibre. However, both of these are eclipsed by the primary solvent in the system, water, which is approximately 76% of the fibroin^57^, and 86% of the sericin^75^, feedstock. Using this approach, assuming that the fluids remain immiscible and that plug flow provides a relevant model, we can substitute the flow as a single phase, consisting solely of the lubricant in order to estimate the reduction in pressure required for flow through lubrication by sericin or water. It was found that the use of sericin^75^, which Kataoka described as a Newtonian fluid, ~10^3^× less viscous than fibroin, still did not reach the spinning domain (See Fig. 8). It is evident that only by using Newtonian water (~10^6^× less viscous) that we begin to enter the domain, yet the upper limits for spinning speed remain inaccessible.

### Experimental validation

Through the incorporation of experimentally derived biological constraints — including a pressure limit, spinning speeds and viscosity variations — our model has clearly indicated that it is not possible to achieve natural spinning by pushing alone. To further validate our simulation, we conducted a series of tests to both measure the forces required to extrude native silk feedstock and to pull a fibre from the animal.

**Fig. 9:**
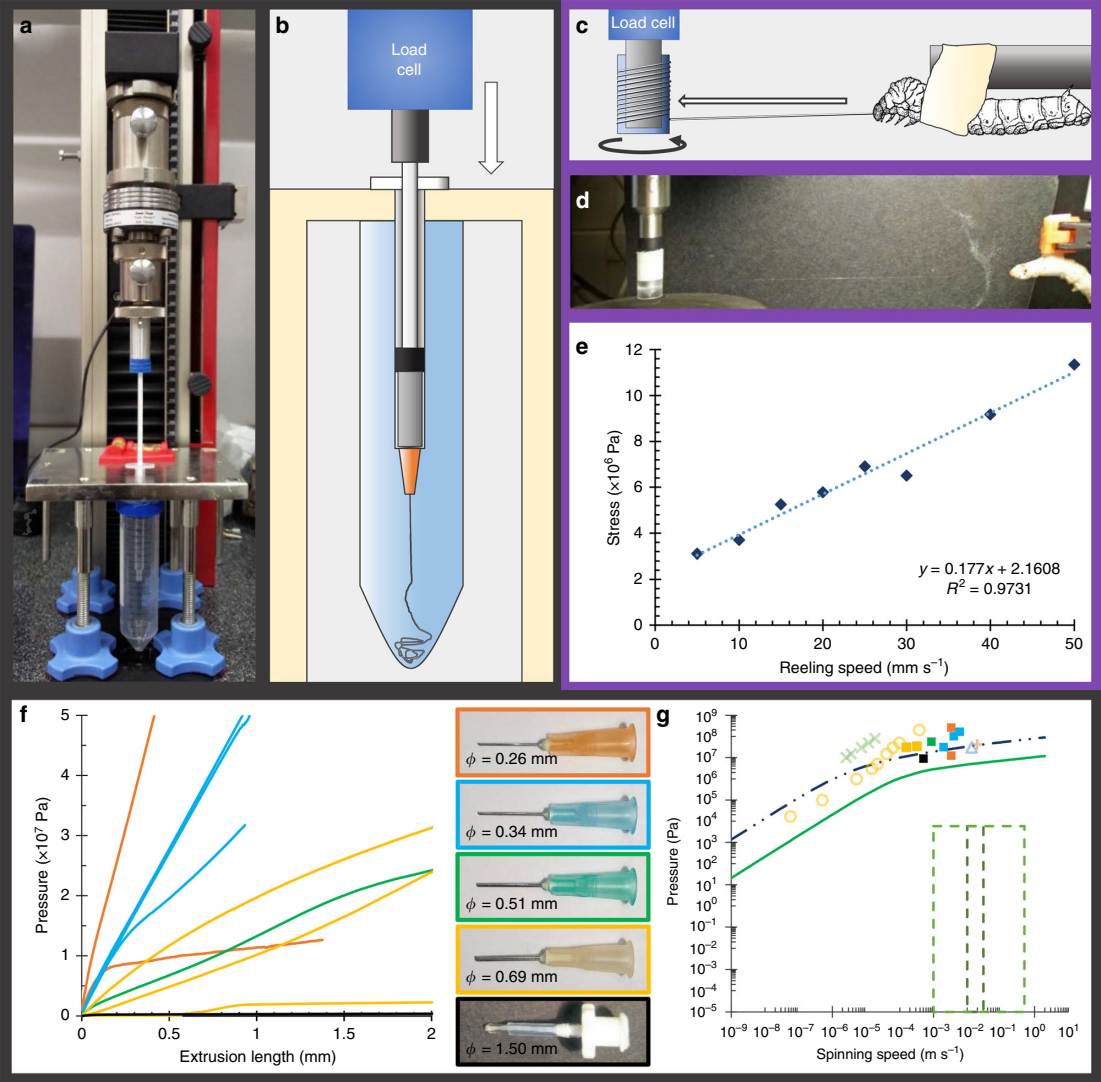
Experimental validation through extrusion of native silk feedstock and pultruding silk fibres via forced reeling. **a** Pushing experimental validation rig: Zwick Z0.5 universal testing machine in compression mode, with hypodermic needle mount and spin tank. **b** Schematic representation of extrusion device seen in **a**. **c** Schematic representing the key components of the forced reeling system (custom geometry/spool and restrained silkworm) used for pultrusion experimental validation. **d** Take-up spool and silkworm mid-reeling experiment, with a strand of silk visible between the two. **e** Plotting stress against reeling speed shows an approximately linear relation between the two variables. The stress encountered during forced reeling is of the same order of magnitude (3–12 MPa) as the pressure predicted through our computational models (6–13 MPa), which supports the argument for pultrusion as the dominant spinning mechanism. **f** The relationship between the pressure required to extrude native silk feedstock through a non-tapering duct and its diameter is shown. *Coloured lines* **f** and *filled squares* **g** refer to the correspondingly coloured hypodermic needles used as extrusion dies. The internal diameter of each is constant over a 20 mm length, and is provided for each size. Values presented are maximum pressures attained. For lower speeds and larger diameters, the measured forces showed an initial rise to a steady plateau where the sample continued to flow through the needle, the value of which is presented in these cases. In the higher speed and/or lower diameter cases, failure during the initial rise in pressure was often experienced, and hence maximum pressure reached is presented as an indicator of minimum pressure required, though it is likely the plateau region would be higher. For clarity, only the first 2 mm of the extruded length are shown, for an overview of the entire extrusion process, see Supplementary Fig. 4. **g** Comparison of our experimental data with previous simulations (Kojic (2006), *orange* *plus*; Moriya (2008), *yellow circles*; Moriya (2009), *green x*; Breslauer (2009), *blue triangle*), our extension of Kojic’s work (*blue dot-dashes*), and the limiting case of our geometric explorations (*pale green*) (see Fig. 5) shows good agreement, with pressures of similar magnitudes to those predicted through simulation required to extrude native silk feedstock through a non-tapering duct

To assess the validity of the effects of geometric variation in our extrusion models, we extracted native fibroin feedstock and subjected it to extrusion tests designed to mimic the limiting case of a 20 mm zero taper duct (Fig. 9a–d). The diameters of the ducts used ranged from 1.5 mm down to 0.26 mm. This lower limit was set by the high probability of Luer-slip connection failure or the plunger critical load *P*_cr_ being surpassed when tests were attempted using smaller diameter ducts, but since both *P*_cr_ (equivalent to ~2.4 × 10^6^ Pa) and the Luer-slip failure pressure (ISO minimum: 3 × 10^5^ Pa) are several orders of magnitude higher than the previously determined biological limit (a rupture pressure of 5.9 × 10^3^ Pa), it seemed unnecessary to explore beyond this.

Experimental results are shown in Fig. 9e–g. Despite the duct diameters being ×10–30 larger than the natural system, the results are in broad agreement with previously published values, and our analysis of duct geometry and fibroin viscosity. These results confirm both that flow in smaller diameter ducts requires greater pressure, and that our simulations are predicting pressure requirements of the correct orders of magnitude.

Given that the predicted and measured forces required to push silk appear to be well in excess of the animal’s biological limits, we are left with the consideration that silk is spun by being pulled. To support this hypothesis, we conducted a series of forced reeling experiments which allowed us to measure the normal stress (in the axial direction) required to forcibly pull fibres from a silkworm, and compare it to the axial stress (i.e., pressure) from our simulation and extrusion results (Fig. 9e).

Figure 9e highlights the stress-reeling speed relation for a representative specimen, which shows not only a strong linear correlation, but also, and of most interest, that silk production across this range of speeds requires stresses of magnitude 3–12 MPa, which both agrees with and further validates our simulation results of 6–13 MPa (Fig. 8). Hence our results show that the forces required to extrude silk feedstock in silico/vitro are in agreement with those achieved through pultrusion in vivo and when pulled can clearly be generated by the silkworm. Therefore, we can conclude that whilst *B. mori* may not have the natural capacity to extrude silk feedstock into fibres, it is capable of generating forces of this magnitude through pultrusion.

## Discussion

Through the use of computational models and experimental validation, we have demonstrated that previously reported requirements for extreme pressure to induce silk flow can be reduced through a variety of factors, which furthers our understanding of the natural system and future bio inspired endeavours.

The first factor involves reducing feedstock viscosity, yet even reducing this a million-fold (to that of water) proved insufficient to access the entirety of the biological extrusion domain. Second, changing the geometry employed, either by increasing outlet diameter or by lessening the severity of the taper, serves to reduce the required pressure. However, the reinforced nature of the tapered section of many silk ducts^72^ suggests that the internal structure is critical, whereas the use of a larger outlet is clearly unrepresentative of the natural system, since spun fibres are much smaller than the diameters required. The final factor considered involves reducing wall friction, with a slip regime used to model the effect of a lubricating layer in the duct. Yet even if all these means were employed by the animal, how would the internal pressure be generated? Previous studies have suggested mechanisms responsible for the creation of such a pressure, such as peristalsis^97^ or osmotic pressure^98^.

Peristalsis seems a plausible explanation, given its prevalence in natural systems—it is used for fluid ejection/excretion/expectoration by many hard bodied animals, including ants^99^ and spitting spiders^100^, and those with soft bodies such as cephalopods^101^. However, the argument against this is that unlike the above examples, the silkworm’s gland is not lined with musculature^71, 102^, and thus the peristaltic effect would need to be transmitted through the haemocoel onto the entire gland at once from the external musculature of the body. Although this may be appropriate for a simple, linear gland, it seems unlikely that such a whole body contraction could create peristaltic flow, due to the twisted, folded nature of the gland itself, with pressure applied at the haemocoel unable to be directed at specific sections of the gland due to the unsegmented nature of the body cavity (Fig. 1).

Another suggestion is that continuous production of silk proteins in the rear of the gland creates a concentration gradient which drives flow through osmosis. However, this would require continuous protein production to maintain the gradient, which is at odds with the knowledge that protein synthesis rates are reduced to zero during cocoon construction in *B. mori*. This is also substantiated by the knowledge that fibroin concentration increases in the flow direction^46^, in direct contradiction to the principles of osmotic flow.

In summary, we suggest that it seems improbable that there is an active pressure system within silkworms capable of generating the high pressures suggested by the flow models. However, the use of a lubricating layer allows plug flow to be considered, and thus the following generalised silk production flow model is presented:

During flow fibroin proteins first align and after a critical shear rate and cumulative stress is exceeded, denature and aggregate together. This results in a phase separation and drives water to the edges of the duct. Here it forms a lubricating layer between the nascent fibre and the duct walls, allowing the system to be treated here as plug flow and as if it were water. Furthermore, it is suggested that the nascent fibre, initially composed of nano and then microfibrils, exhibits a solidity gradient along its length, in which the fibre increases in modulus as it travels down the gland, allowing fibres to be pulled from the animal by themselves.

Of course, these push/pull effects are not mutually exclusive, and thus several could be in use within the system. This means that, although we cannot conclusively say that there is no pushing in the system, we can state with certainty that it cannot be considered as the dominant force acting within the system, and that instead, our data show the maximum rates achievable through pulling alone, hence the difference between this and the natural system represents a minimum force requirement for pultrusion. The next step in the process is to develop multiphase models, requiring the determination of the rheological properties of fluids such as sericin. In conclusion, this study represents a step forward in our understanding of the conditions by which natural spinning occurs and identifies the requirement for the development of biomimetic artificial silk spinning devices based on pultrusion rather than extrusion.

## Methods

### Experimental

*Extrusion*: Simulations were validated through two experimental studies. To assess the validity of extrusion in generating sufficient flow rates, native silk fibroin feedstock from the middle and anterior portion of the silk gland was extracted as described elsewhere^57^ and loaded into 1.0 ml syringes treated with a super hydrophobic coating (Rain-x) for ease of loading. A modified universal testing machine (Zwick Roell, Z0.5, 500 N load cell) was used to provide the back pressure to flow the feedstock through 20 mm needles (diameters from 0.26 to 1.5 mm) into a water bath. Spinning speeds between 0.16 and 6.04 mm s^−1^ were successfully applied to 10 specimens. However, when higher rates were applied, either the Luer connection between syringe and needle failed, or the Euler load of the plunger was exceeded, both of which invalidated the test.

*Forced reeling*: Final instar *B. mori* silkworms in their wandering phase were force reeled using a rheometer as a high sensitivity force transducer capable of much longer reeling times (effectively indefinite) than previous work^22, 25^. A total of 10 silkworms were reeled across a range of spinning speeds (5–75 mm s^−1^). Although the possible range of forced reeling rates is broader than this, for ethical reasons we did not explore the full range reported in the literature (1–500 mm s^−1^)^15, 18, 21, 22, 24–28^ due to the requirement of paralysis for low rates and the potential for permanent damage to the animal at higher rates. Average fibre diameters from silkworms were determined from between 77 and 295 (dependent on quantity of silk reeled) independent measurements from photos taken through a reflection microscope under ×20 magnification (lasertec E414) and quantified using ImageJ.

### Simulation

*Overview*: Finite element analysis of flow within silk ducts was performed using the computational fluid dynamics (CFD) element of the COMSOL Multiphysics 5.2 software package. Simulations have been run in an isothermal environment with ambient external pressure, both of which are considered representative of the natural system.

*Geometry*: Ducts have been modelled using geometric approximations of experimentally determined data^42, 71, 72, 87^, as seen previously^43, 71^. This is considered a suitable technique to determine the system geometry as it provides a more rigorous, general description than that utilised in other FE studies^40, 73^. The system has been simplified to a two-dimensional, axisymmetric representation for computational efficiency. Wall conditions have been modelled in the limiting cases of both no-slip and full-slip boundaries in order to fully capture the domain of interest.

*Mesh*: Models were repeatedly re-meshed to determine mesh convergence, resulting in systems of ~20,000 elements, which provides the optimum trade-off between computational accuracy and time, with the variation in results ~0.02% when compared with models comprising 15 million elements.

*Flow regime*: The flow is assumed to be laminar (taking maximum values for density, velocity and diameter, and minimum viscosity yields Reynolds numbers in the region of Re < 10), and is modelled as a single fluid phase (in the absence of data describing the rheological properties of sericin (an area of current research) we have assumed this to be the best case).

*Density and concentration*: The density of silk feedstock has often been misrepresented in the literature, with previous studies treating spun and unspun density as equivalent^40, 73, 103^—this is unrepresentative due to the dehydrated, solid, state of spun silks. Later studies attempted to account for this but were held back by smaller sample sizes, resulting in a 25% overshoot^43, 104^. A closer approximation can be made from a mass fraction calculation assuming 24% (s.d. = 2.5%) concentration^57^ to arrive at a density of 1072 kg m^−3^.

The effect of concentration has not been included beyond the effect it has on fluid density due to its unexpectedly weak correlation with viscosity^57, 58^. Furthermore the proposed phase separation and water resorption^105^ in the distal regions are ignored as recent evidence has suggested that silk may be more hydrated as it exits the animal than has been reported previously^106^.

*Spinning speed*: Spinning speeds have been analysed from 1 × 10^−9^ m s^−1^ to 2 m s^−1^, which encompasses all verifiable previous recorded spinning speeds, forced or natural^15, 18,21, 22, 24–28^. The single inlet/outlet model ensures that mass is conserved, thus we can use exit velocity (spinning speed) to determine inlet conditions, in this case specified by the pressure.

### Data availability

The data sets generated during and/or analysed during the current study are available in the University of Sheffield’s Online Research Data Archive (ORDA) repository, http://dx.doi.org/10.15131/shef.data.4990916.

## Electronic supplementary material

Supplementary Information

Peer Review
