# Supplementary material for: Analysis of the pressure requirements for silk spinning reveals a pultrusion dominated process
Source: Nat Commun. 2017 Sep 19;8:594. doi: 10.1038/s41467-017-00409-7 (PMC5605702; doi:10.1038/s41467-017-00409-7)
Supplement: Supplementary file 1 — Supplementary Information [file 41467_2017_409_MOESM1_ESM.pdf]

Description of Supplementary Files

File Name: Supplementary Information
Description: Supplementary Figures and Supplementary Tables

File Name: Peer Review File

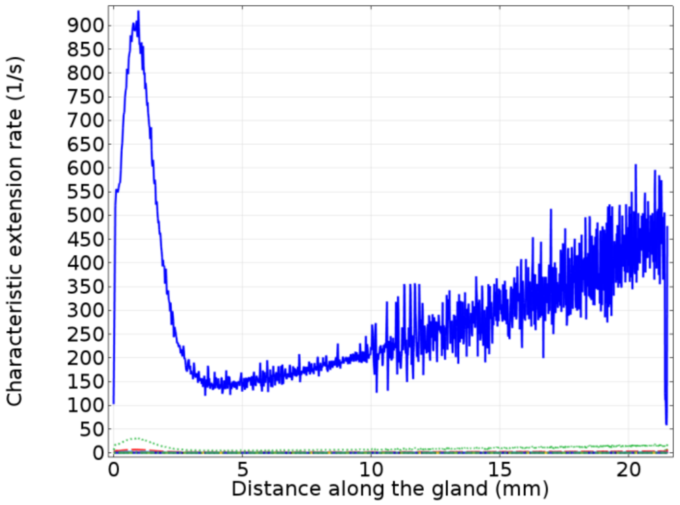

Supplementary Figure 1: variation in extension rates along the gland.

*Variation in extension rates along the silkworm gland are readily observed across a range of zero-shear viscosities. The primary data set shown here is for a zero-shear viscosity of 500Pa.s, but while the magnitude changes for different zero-shear viscosities, the general trend remains the same. Simulation performed using Breslauer's hyperbolic geometry (see **Supplementary Table 1**).*

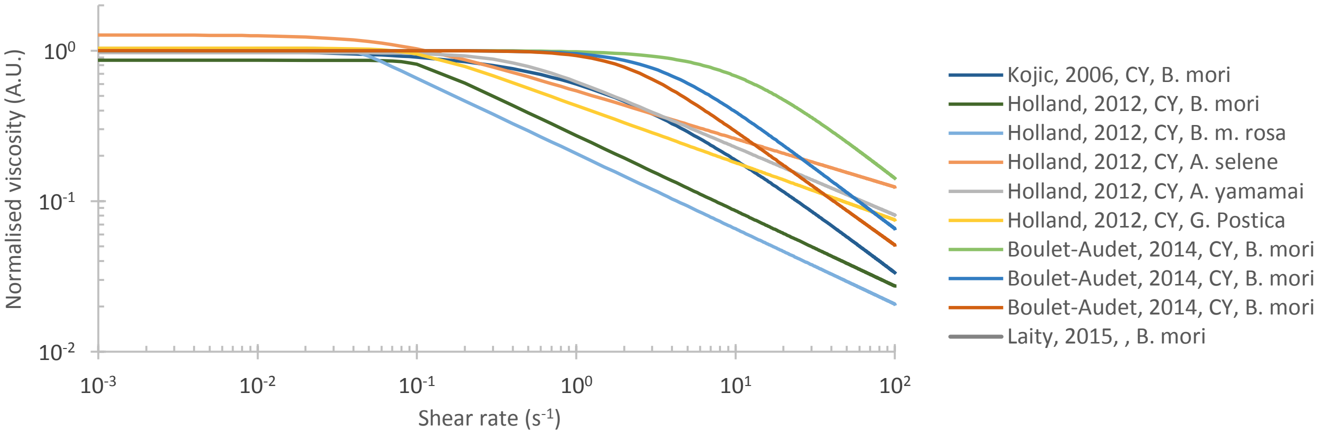

Supplementary Figure 2: Viscosity models normalised against zero shear viscosity

Viscosity models normalised against zero shear viscosity for B. mori and wild silkworms shows that despite the wide range of zero shear viscosities encountered, the rate of shear thinning is similar. [Key details: Author, year, viscosity model, species]

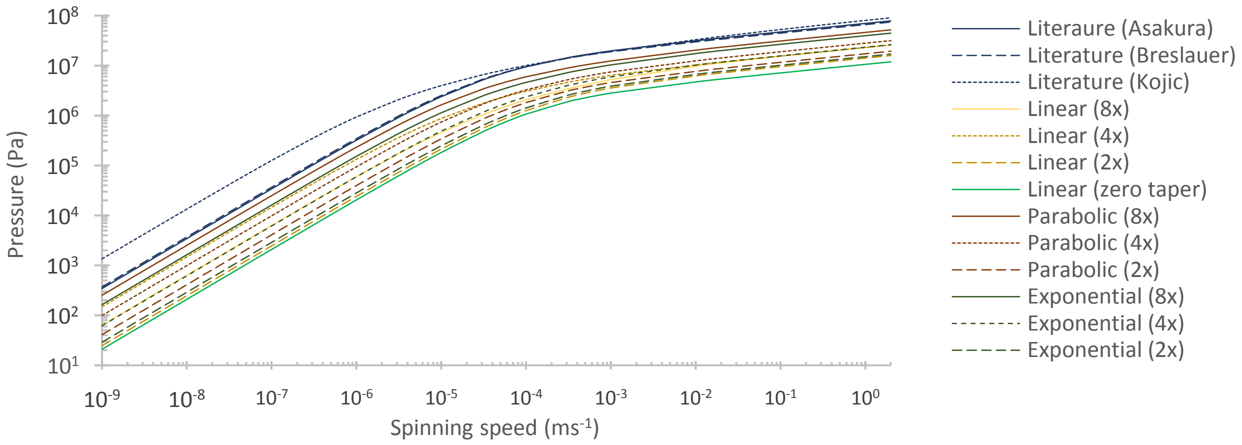

Supplementary Figure 3 - Full data set for geometric variation

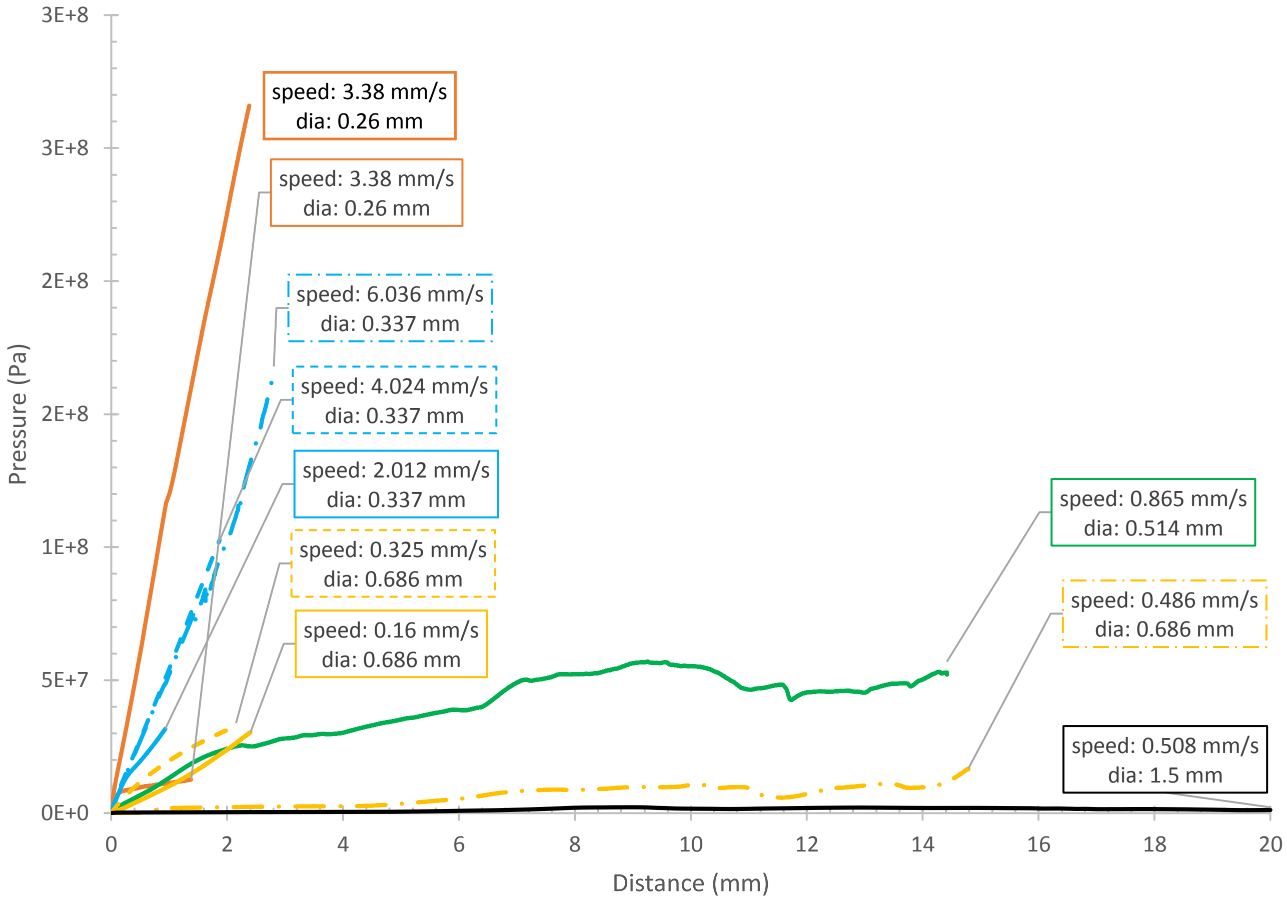

Supplementary Figure 4: Complete version of Experimental validation extrusion tests (Figure 9f).

The relationship between pressure required to extrude native silk dope and the diameter of the spinning duct used is shown here. For larger diameter ducts, an initial rise in force required leads to a steady plateau and extrusion of the entirety of the dope used. (Black, 1.5mm; yellow, 0.69mm, green, 0.51mm). However, the initial rise in pressure for narrow ducts (blue, 0.34mm; orange, 0.26mm) exceeds the luer-slip limit and/or the critical load for plunger buckling and hence the plateau region is not reached in these cases. However the general trend in the early stages of the experiment is clear from the figure in the main text.

Supplementary Table 1 - Mathematical functions used to approximate the duct geometry in previous studies

Breslauer	$r(z) = ae^{bz} + ce^{dz}$	a=1.398 × 10^2 µm<br>b=1.97 × 10^{-2} µm^{-1}<br>c=5.845 x 10^1 µm<br>d=-3.58 × 10^{-5} µm^{-1}
Asakura	$r(z) = a\left(\frac{1}{1+e^{bz}}\right) + c(\frac{1}{1+ e^{dz}})$	a =2.38 × 10^2 µm<br>b=6.18 × 10^{-5} µm^{-1}<br>c= 5.88 × 10^2 µm<br>d=3.00 × 10^{-3} µm^{-1}
Kojic	$r(z) = az + b$	a = -4.9 × 10^{-3}<br>b=2 x 10^2 µm

Supplementary Table 2 - Mathematical functions used to approximate the duct geometry in this study.

Type of taper	Equation	(values in m or m^{-1} as appropriate)			
		12.5% of inlet	25% of inlet	50% of inlet	100% of inlet
Linear	$r(z) = az + b$	a = -8.44 x 10^{-3}<br>b =2.065 x 10^{-4}	a = -7.28 x 10^{-3}<br>b =2.065 x 10^{-4}	a = - 4.95 x 10^{-3}<br>b =2.065 x 10^{-4}	a = 0<br>b = 2.065 x 10^{-4}
Exponential	$r(z) = ae^{bz}$	a = 2.065 x 10^{-4}<br>b = -9.836 x 10^1	a = 2.065 x 10^{-4}<br>b = - 6.597 x 10^1	a = 2.065 x 10^{-4}<br>b = -3.373 x 10^1	Not applicable
Parabolic	$r(z) = az^2 + bz + c$	a = 3.928 x 10^{-1}<br>b = -1.69 x 10^{-2}<br>c = 2.065 x 10^{-4}	a = 3.386 x 10^{-1}<br>b = -1.46 x 10^{-2}<br>c = 2.065 x 10^{-4}	a = 2.304 x 10^{-1}<br>b = -9.9 x 10^{-3}<br>c = 2.065 x 10^{-4}	Not applicable
