# Supplementary material for: Analysis of the pressure requirements for silk spinning reveals a pultrusion dominated process
Source: Nat Commun. 2017 Sep 19;8:594. doi: 10.1038/s41467-017-00409-7 (PMC5605702; doi:10.1038/s41467-017-00409-7)
Supplement: Supplementary file 2 — Peer Review [file 41467_2017_409_MOESM2_ESM.pdf]

Reviewers' comments:

Reviewer #1 (Remarks to the Author):

This is a very well written and detailed study on the fibre spinning of silk fibroin using a computational model and I applaud the authors for taking a well thought approach in the development of the COMSOL model. Silk fibre spinning is still challenging and biomimetic devices to date haven't been able to produce fibres that are comparable to native fibres. The development of the model is based on several earlier studies that provide the basis for the models. The COMSOL model provides fodder for development of artificial silk spinning devices. As such this is only a model and would require experimental validation.

Technically I have no issues with the paper but would suggest that either the authors revise this manuscript with experimental data to validate their predictive models or submit to a more specialized journal. Experimental validation is critical for impact of this study and as such I am skeptical till experiments are carried out.

Reviewer #2 (Remarks to the Author):

The authors have used finite element simulations to explore the role of internal pressure, spinning duct geometry, zero shear viscosity and wall friction on the flow rate of the generated fiber. From their results they conclude that silk being pulled out of the duct plays a significant role in the mechanism of silk formation. The paper is well written and easy to read with a comprehensive list of references. The arguments are all plausible bar the effect of uniform pressure on the gland.I have made some, mostly minor, comments directly on the manuscript pdf.

Reviewer #3 (Remarks to the Author):

The paper is well written and based on the authors' approach. It seems that spinning is achieved by pulling as opposed to pushing.
However, the paper could be improved if two topics were discussed in more detail:
a- If both mechanisms –push and pulling– actuated simultaneously, will there be any difference on the pushing pressure?
b- More information on how the silkworm is pulling the fiber would be welcome.

Additional information on the boundaries of the biomimetic spinning domain would also be welcome, particularly on available realistic spinning speeds.

Some passages would benefit from explanation in more detail, such as (lines 110-116) and (lines 275-281).

Minor comments:
Add a reference after B mori (line 197)
It would be helpful when comparing text and figures (and also in the same figure, as Fig. 5) to use always the same notation, instead of (Pa, kPa, MPa, 10x Pa) or (nms-1, ms-1,10-xms-1)

Reviewer #1

Comment: |This is a very well written and detailed study on the fibre spinning of silk fibroin using a computational model and I applaud the authors for taking a well thought approach in the development of the COMSOL model. Silk fibre spinning is still challenging and biomimetic devices to date haven't been able to produce fibres that are comparable to native fibres. The development of the model is based on several earlier studies that provide the basis for the models. The COMSOL model provides fodder for development of artificial silk spinning devices. As such this is only a model and would require experimental validation.

Technically I have no issues with the paper but would suggest that either the authors revise this manuscript with experimental data to validate their predictive models or submit to a more specialized journal. Experimental validation is critical for impact of this study and as such I am sceptical till experiments are carried out.

We thank the reviewer for their kind comments. With regard to their request to see experimental validation to widen the impact, we have now provided further references to several previous studies in our discussion to better support our findings. See line 82 (Knight & Vollrath 1999; Asakura et al. 2007; Davies et al. 2013b; Davies et al. 2013a), and lines 105-107 (Kataoka & Uematsu 1977; Terry et al. 2004; Moriya et al. 2009; Ochi et al. 2002; Holland et al. 2012; Holland et al. 2006; Laity et al. 2015; Laity & Holland 2016; Moriya et al. 2008; Kojic et al. 2006; Boulet-Audet et al. 2014), and

---

Reviewer #2:

Comment: |The authors have used finite element simulations to explore the role of internal pressure, spinning duct geometry, zero shear viscosity and wall friction on the flow rate of the generated fibre. From their results they conclude that silk being pulled out of the duct plays a significant role in the mechanism of silk formation. The paper is well written and easy to read with a comprehensive list of references.

Response: |We thank the reviewer for their positive appraisal of our work.

Comment: | The arguments are all plausible bar the effect of uniform pressure on the gland.

Response: |L273 – we apologise for the lack of clarity, and have re-worded this statement to discuss the likelihood (or lack thereof) of peristalsis being a method for transportation within the gland. See Lines 273-282

> *and thus the peristaltic effect would need to be transmitted through the haemocoel onto the entire gland at once from the external musculature of the body. Although this may be appropriate for a simple, linear gland, it seems unlikely that such a contraction could create peristaltic flow, due to the twisted, folded nature of the gland itself, with pressure applied at the haemocoel unable to be directed at specific sections of the gland due to the unsegmented nature of the body cavity. Another common suggestion is that continuous production of silk proteins in the rear of the gland creates a concentration gradient which drives flow through osmosis. However, this system would require production throughout the spinning process, which is at odds with the single spinning event that occurs post-production in B. mori, and suggests that this is not an appropriate mechanism for driving flow. This is substantiated by the knowledge that fibroin concentration increases in the flow direction (Percot et al. 2014), in direct contradiction to the principles of osmotic flow.*

Comment: |I have made some, mostly minor, comments directly on the manuscript pdf.

Response: |L8 – we have changed explore to review.

Response: |Lines 121-123: the lack of dots/dashes in the figures is regrettably our mistake and an artefact of the PDF conversion process. These have been updated accordingly.

Response: |L134 – use of word models: We agree that this was poorly phrased and have altered this to be clearer.

Now lines 133-136:

> *The simplified form of the CY-model allows the viscosity to be described in terms of its zero shear viscosity, (readily estimated from low shear measurements, and the rate of shear thinning.) which seems appropriate, given that the primary difference between individual specimens lies in the reported values of the zero-shear viscosity*

Response: |Lines 214 to 233 – Unclear labelling in figure 6 and 7. We have removed the superfluous arrows, replacing them with clear labels and provided further details in the caption. See lines 217-219

> *Figure 1 - The effect of varying the zero shear viscosity (across the range 400-5000 Pa.s*(Laity et al. 2015) *(orange), mean (1722 Pa.s*(Laity & Holland 2016)*, green), and 200-10000 Pa.s*(Laity & Holland 2016) *(blue),) on the required inlet pressure. Since geometry*

was earlier shown to have little effect on the pressure requirements, this study employed the same geometry as Breslauer(Breslauer et al. 2009) *to allow comparison with previously published work. The biomimetic spinning domain is split into natural (dark green) and forced (light green) spinning domains.*

And lines 236-238

Figure 2 – The assumption of a slip condition at the duct wall (with the same geometric and rheological data as used in Fig. 1) brings the lower portion of Laity's ranges (green, mean-1722 Pa.s(Laity et al. 2015; Laity & Holland 2016)*, Orange, 400-5000 Pa.s*(Laity & Holland 2016)*, Blue 200-10000 Pa.s*(Laity & Holland 2016)*) into the biomimetic spinning domain. The biomimetic spinning domain is split into natural (dark green) and forced (light green) spinning domains.*

Reviewer #3

Comment: |The paper is well written and based on the authors' approach. It seems that spinning is achieved by pulling as opposed to pushing.

Response*:* |We would like to take the opportunity to thanks the reviewer for their detailed feedback.

Comment: |However, the paper could be improved if two topics were discussed in more detail: If both mechanisms – push and pulling– actuated simultaneously, will there be any difference on the pushing pressure?

Response*:* | This is a really interesting point which we are glad to have been given the opportunity to raise in the revised manuscript. As a result we have added the following paragraphs and references to Lines 294-298 in the manuscript.

Of course, these effects are not mutually exclusive, and thus several could be in use within the system. This means that, although we cannot conclusively write off pushing in the system, we can state with certainty that it cannot be considered as the dominant force acting within the system, and that instead, our data shows the maximum rates achievable through pulling alone, hence the difference between this and the natural system represents a minimum force requirement for pultrusion.

Comment: |More information on how the silkworm is pulling the fibre would be welcome.

Response*:* |We are happy to do so. We have now added the following text to Lines 290-292 in the manuscript.

allowing the fibres to be pulled by the worm from itself against their anchorage (either the cocoon site or internal cocoon wall, depending on the extent of cocoon construction).

Comment: |Additional information on the boundaries of the biomimetic spinning domain would also be welcome, particularly on available realistic spinning speeds.

Response*:* |We apologise for this omission and have now included values and references in text for spinning speeds to supplement those already listed for pressures. Lines 177-178

An initial constraint is the range of natural spinning speeds (0.01 – 0.03 ms-1)(Mortimer et al. 2013; Shao & Vollrath 2002)*, but we widen this to incorporate forced reeling (0.001 – 0.5 ms-1)*

Comment: |Some passages would benefit from explanation in more detail, such as (lines 110-116) and (lines 275-281).

Response*:* |We have now clarified the passages on viscoelastic models (Line 110-116), with greater reference to specific regions within figure 2b, and completely rewritten that discussing osmosis (Line 275-281). Now Lines 111-117

The mathematical models that have been used to describe the viscoelastic behaviour of fibroin are both complex and not well defined, particularly at higher shear rates where fibre formation is known to begin. Early attempts to describe this behaviour used a different power law functions to describe each of phases 1-4 figure 2b (Ochi et al. 2002; Terry et al. 2004; Moriya et al. 2008; Moriya et al. 2009)*. However, as we are not concerned with the fibrillation region figure 2b, regions 3 and 4) (a biomimetic design limit – premature fibrillation will be naturally selected against, as blockages would prevent further production), models which describe the behaviour at shear rates below this point (regions 1 and 2 in figure 2**Error! Reference source not found.**b) are considered adequate. As such, the Carreau-Yasuda model*(Yasuda et al. 1981) *(equation 1) is now the preferred model as it describes regions 1 and 2 in a single equation.*

And Lines 273- 282

"and thus the peristaltic effect would need to be transmitted through the haemocoel onto the entire gland at once from the external musculature of the body. Although this may be appropriate for a simple, linear gland, it seems unlikely that such a contraction could create peristaltic flow, due to the twisted, folded nature of the gland itself, with pressure applied at the haemocoel unable to be directed at specific sections of the gland due to the unsegmented nature of the body cavity. Another suggestion is that continuous production of silk proteins in the rear of the gland creates a concentration gradient which drives flow through osmosis. However, this system would require production throughout the spinning process, which is at odds with the single spinning event that occurs post-production in B. mori, and suggests that this is not an appropriate mechanism for driving flow. This is substantiated by the knowledge that fibroin concentration increases in the flow direction(Percot et al. 2014)*, in direct contradiction to the principles of osmotic flow. "*

Comment: |Add a reference after B mori (line 197)

Response*:* |We have added in specific references at line 198 (Moreau 1974; Prakash 2008) for this statement.

Comment: |It would be helpful when comparing text and figures (and also in the same figure, as Fig. 5) to use always the same notation, instead of (Pa, kPa, MPa, 10x Pa) or (nms-1, ms-1, 10-xms-1)

Response*:* |We thank the reviewers for highlighting this discrepancy, we have corrected our manuscript to provide consistent units of metres and Pascals.

References

Asakura, T. et al., 2007. Some observations on the structure and function of the spinning apparatus in the silkworm Bombyx mori. *Biomacromolecules*, 8(1), pp.175–181.

Boulet-Audet, M. et al., 2014. Silk protein aggregation kinetics revealed by Rheo-IR. *Acta Biomaterialia*, 10(2), pp.776–784. Available at: http://dx.doi.org/10.1016/j.actbio.2013.10.032.

Breslauer, D.N., Lee, L.P. & Muller, S.J., 2009. Simulation of Flow in the Silk Gland. *Biomacromolecules*, 10(1), pp.49–57. Available at: http://pubs.acs.org/doi/abs/10.1021/bm800752x.

Davies, G.J.G., Knight, D.P. & Vollrath, F., 2013a. Chitin in the silk gland ducts of the spider Nephila edulis and the silkworm Bombyx mori. *PLoS ONE*, 8(8), p.e73225.

Davies, G.J.G., Knight, D.P. & Vollrath, F., 2013b. Structure and function of the major ampullate spinning duct of the golden orb weaver, Nephila edulis. *Tissue and Cell*, 45(5), pp.306–311. Available at: http://dx.doi.org/10.1016/j.tice.2013.04.001.

Holland, C. et al., 2006. Comparing the rheology of native spider and silkworm spinning dope. *Nature materials*, 5(11), pp.870–874.

Holland, C., Porter, D. & Vollrath, F., 2012. Comparing the rheology of mulberry and “wild” silkworm spinning dopes. *Biopolymers*, 97(6), pp.362–367.

Kataoka, K. & Uematsu, I., 1977. Studies on liquid silk VI - on the viscosity of liquid silk in the silkgland. *Kobunshi Ronbunshu*, 34(1), pp.7–13. Available at: http://joi.jlc.jst.go.jp/JST.Journalarchive/koron1974/34.7?from=CrossRef.

Knight, D.P. & Vollrath, F., 1999. Liquid crystals and flow elongation in a spider ’s silk production line. *Proceedings of the Royal Society London*, 266(November 1998), pp.519–523. Available at: http://rspb.royalsocietypublishing.org/content/266/1418/519.short.

Kojic, N. et al., 2006. Ex vivo rheology of spider silk. *The Journal of experimental biology*, 209(Pt 21), pp.4355–4362.

Laity, P.R., Gilks, S.E. & Holland, C., 2015. Rheological behaviour of native silk feedstocks. *Polymer*, 67, pp.28–39. Available at: http://linkinghub.elsevier.com/retrieve/pii/S0032386115003869.

Laity, P.R. & Holland, C., 2016. Native Silk Feedstock as a Model Biopolymer: A Rheological Perspective. *Biomacromolecules*, 17(8), pp.2662–2671. Available at: http://pubs.acs.org/doi/abs/10.1021/acs.biomac.6b00709.

Moreau, R., 1974. Variations de la pression interne au cours de l’émergence et de l’expansion des ailes chez Bombyx mori et Pieris brassicae. *Journal of Insect Physiology*, 20(8), pp.1475–1480.

Moriya, M. et al., 2008. Flow analysis of aqueous solution of silk fibroin in the spinneret of Bombyx mori silkworm by combination of viscosity measurement and finite element method calculation. *Polymer*, 49(4), pp.952–956.

Moriya, M. et al., 2009. Rheological properties of native silk fibroins from domestic and wild silkworms, and flow analysis in each spinneret by a finite element method. *Biomacromolecules*, 10(4), pp.929–935.

Mortimer, B., Holland, C. & Vollrath, F., 2013. Forced reeling of bombyx mori silk: Separating behavior and processing conditions. *Biomacromolecules*, 14, pp.3653–3659.

Ochi, A. et al., 2002. Rheology and dynamic light scattering of silk fibroin solution extracted from the middle division of Bombyx mori silkworm. *Biomacromolecules*, 3(6), pp.1187–1196.

Percot, A. et al., 2014. Water dependent structural changes of silk from Bombyx mori gland to fibre as evidenced by Raman and IR spectroscopies. *Vibrational Spectroscopy*, 73, pp.79–89. Available at: http://dx.doi.org/10.1016/j.vibspec.2014.05.004.

Prakash, M., 2008. *Encyclopaedia of Entomology - III. Insect physiology* 1st ed., Ghaziabad: Discovery Publishing House.

Shao, Z. & Vollrath, F., 2002. Surprising strength of silkworm silk. *Nature*, 418(6899), p.741.

Terry, A.E. et al., 2004. pH induced changes in the rheology of silk fibroin solution from the middle division of Bombyx mori silkworm. *Biomacromolecules*, 5(3), pp.768–772.

Yasuda, K., Armstrong, R.C. & Cohen, R.E., 1981. Shear flow properties of concentrated solutions of linear and star branched polystyrenes. *Rheologica Acta*, 20(2), pp.163–178.

Reviewers' comments:

Reviewer #1 (Remarks to the Author):

I would prefer to see experimental validation rather then references to support the model.

Reviewer #2 (Remarks to the Author):

I am happy that the changes made have addressed my earlier concerns

Reviewer #3 (Remarks to the Author):

No remarks

Response to reviewers' comments:

Reviewer #1 *(Remarks to the Author):*

Comment: | I would prefer to see experimental validation rather than references to support the model.

We thank the reviewer for their consideration of our manuscript. Whilst we originally believed that reference to previous studies were sufficient to support our simulation, upon reflection and discussion we are genuinely grateful for the reviewer's comment, as it has led us towards what we feel is a more rigorous approach to the topic.

We have spent the past few months conducting validation experiments detailed in the new subsection titled "experimental validation". In order to validate our simulation work, we have considered the twin aspects of the question we posed as to whether silk is pushed or pulled, and have developed experiments which approach this problem from both the perspective of pushing, and of pulling.

We are pleased to report that both new methodologies have provided evidence which supports our hypothesis. The extrusion rig has demonstrated that the pressures required are both far in excess of what silkworms have the capacity to generate, and are also in line with both our, and previous, simulation studies. Our reeling rig has provided more support for our closing hypothesis, showing that through pultrusion, it is possible to generate stresses that are required to spin fibres in the same order of magnitude as those predicted through our simulations.

We believe both experimental approaches, in their non-typical use of a tensile testing machine and a rheometer as measurement devices, display experimental novelty that is in itself useful to the field, opening doors for further exploration. For the extrusion device we believe it may aid those in developing silk feedstock spinning devices. In the case of the rheometer-reeling device we can now perform practically unlimited reeling with greater control over processing conditions than the previous systems such as those used by Mortimer (Mortimer et al. 2013; Mortimer et al. 2015).

In summary we hope that our new experimental section broadens the impact and novelty of the manuscript and that we have addressed any concerns the reviewer may have held about the validity of simulation work.

Reviewer #2 *(Remarks to the Author):*

Comment: | I am happy that the changes made have addressed my earlier concerns

We thank the reviewer for their support of our work, and are pleased that we have adequately addressed their concerns.

Reviewer #3 *(Remarks to the Author):*

Comment: | No remarks

We thank the reviewer for time on this manuscript.

REVIEWERS' COMMENTS:

Reviewer #1 (Remarks to the Author):

I appreciate the authors performing the experiments to support the modeling work, and makes the manuscript much stronger. I recommend acceptance of manuscript.
